# Construyo e validado de vídeo sobre o cáncer de mama para surdas*

**DOI:** 10.15649/cuidarte.3076

**Published:** 2023-12-24

**Authors:** Júlia Diana Pereira-Gomes, Aline Tomaz-De-Carvalho, Maria Girlane Sousa-Albuquerque-Brandao, Nelson Miguel Galindo-Neto, Maria Do-Livramento-Fortes-Figueiredo, Monaliza Ribeiro Mariano-Grimaldi

**Affiliations:** 1 . Universidade da Integrado Internacional da Lusofonia Afro-Brasileira, Redenáo, Brasil. Email: juliagomes.jd@gmail.com Universidade Federal da Integração Internacional da Lusofonia Afro-Brasileira Universidade da Integrado Internacional da Lusofonia Afro-Brasileira Redenáo Brazil juliagomes.jd@gmail.com; 2 . Unimed Fortaleza, Fortaleza, Brasil. Email: aline.nurse@gmail.com Unimed Fortaleza Fortaleza Brasil aline.nurse@gmail.com; 3 . Universidade de Sáo Paulo, Sáo Paulo, Brasil. Email: girlanealbuquerque@usp.br Universidade de São Paulo Universidade de Sáo Paulo Sáo Paulo Brazil girlanealbuquerque@usp.br; 4 . Instituto Federal de Educado, Ciencia e Tecnologia de Pernambuco, Recife, Brasil. E-mail: nelsongalindont@hotmail.com Fundação de Amaparo à Ciência e Tecnologia de Pernambuco Instituto Federal de Educado, Ciencia e Tecnologia de Pernambuco Recife Brazil : nelsongalindont@hotmail.com; 5 . Universidade Federal do Piauí, Teresina, Brasil, Email: liff@ufpi.edu.br Universidade Federal do Piauí Universidade Federal do Piauí Teresina Brazil liff@ufpi.edu.br; 6 . Universidade da Integrado Internacional da Lusofonia Afro-Brasileira, Redenáo, Brasil. Email: monalizamariano@unilab.edu.br Universidade da Integração Internacional da Lusofonia Afro-Brasileira Universidade da Integrado Internacional da Lusofonia Afro-Brasileira Redenáo Brazil monalizamariano@unilab.edu.br

**Keywords:** Surdez, Filme e Vídeo Educativo, Neoplasias da Mama, Educado em Saúde, Deafness, Instructional Film and Video, Breast Neoplasms, Health Education, Sordera, Película y Video Educativos, Neoplasias de la Mama, Educación en Salud

## Abstract

**Introdujo:**

No mundo, um bilháo de pessoas tem algum tipo de deficiencia, dentre elas a deficiencia auditiva e mais de 190 milhóes sáo mulheres surdas. É relevante tecnologias para educado inclusiva e acessível sobre o cáncer de mama a surdas.

**Objetivo:**

Construir e validar vídeo educativo para mulheres surdas sobre prevencáo e rastreamento do cáncer de mama.

**Materiais e Método:**

Estudo metodológico com construáo de vídeo educativo e validado por 11 especialistas de conteúdo e sete especialistas técnicos. Conteúdo fundamentado no Ministério da Saúde e no Instituto Nacional do Cáncer. Na análise de conteúdo foi utilizado o Instrumento de Validado de Conteúdo Educativo em Saúde. A validade foi atestada pelo Índice de Validade de Conteúdo e o teste binomial.

**Resultados:**

O vídeo tem 17 minutos e 12 segundos. Na validado de conteúdo todos os itens tiveram concordáncia acima de 90,9% entre os participantes. O Índice de Validade de Conteúdo global foi superior a 0,90 entre os especialistas de conteúdo. Consideraram o material excelente, muito bom ou bom, 85,8% dos especialistas técnicos.

**Discussao:**

O vídeo contribui para o ensino e aprendizagem sobre o tema, aóes de autocuidado e promodo da saúde as surdas.

**Conclusoes:**

Vídeo construído com narrado em LIBRAS, foi considerado válido quanto ao conteúdo e aspectos técnicos e poderá ser utilizado em futuras pesquisas e na educado em saúde de surdas.

## Abstract (en)

**Introduction:**

In the world, one billion people have some kind of disability, among them hearing impairment and more than 190 million are deaf women. It is relevant technologies for inclusive and accessible education about breast cancer to deaf women.

**Objective:**

To build and validate an educational video for deaf women on breast cancer prevention and screening.

**Materials and Method:**

Methodological study with construction of educational video and validation by 11 content specialists and seven technical specialists. Content based on the Ministry of Health and the National Cancer Institute. The Health Education Content Validation Instrument was used for content analysis. Validity was attested by the Content Validity Index and the binomial test.

**Results:**

The video is 17 minutes and 12 seconds long. In the content validation all items had agreement above 90.9% among participants. The overall Content Validity Index was above 0.90 among the content experts. 85.8% of the technical experts considered the material excellent, very good or good.

**Discussion:**

The video contributes to teaching and learning about the theme, self-care actions and health promotion for deaf women.

**Conclusions:**

Video constructed with narration in LIBRAS, was considered valid in terms of content and technical aspects and can be used in future research and health education for deaf women.

## Resumen

**Introducción:**

En el mundo, mil millones de personas tienen algún tipo de discapacidad, entre ellas discapacidad auditiva y más de 190 millones son mujeres sordas. Se trata de tecnologías relevantes para una educación inclusiva y accesible sobre el cáncer de mama para mujeres sordas.

**Objetivo:**

Construir y validar un vídeo educativo para mujeres sordas sobre prevención y cribado del cáncer de mama.

**Materiales y Método:**

Estudio metodológico con construcción de video educativo y validación por 11 especialistas de contenido y siete especialistas técnicos. Contenido basado en el Ministerio de Salud y el Instituto Nacional del Cáncer. Se utilizó la Herramienta de Validación de Contenidos de Educación para la Salud para analizar el contenido. La validez fue atestada por el Índice de Validez de Contenido y la prueba binomial.

**Resultados:**

El vídeo tiene una duración de 17 minutos y 12 segundos. En la validación del contenido todos los ítems tuvieron un acuerdo superior al 90,9% entre los participantes. El Índice de Validez de Contenido global fue superior a 0,90 entre los expertos en contenido. El 85,8% de los expertos técnicos consideraron el material excelente, muy bueno o bueno.

**Discusión:**

El vídeo contribuye a la enseñanza y el aprendizaje sobre el tema, las acciones de autocuidado y la promoción de la salud de las mujeres sordas.

**Conclusiones:**

El video construido con narración en LIBRAS, fue considerado válido en términos de contenido y aspectos técnicos y puede ser utilizado en futuras investigaciones y educación en salud para mujeres sordas.

## Introdujo

O cáncer de mama é uma preocupado mundial devido a sua alta incidencia. Em 2018, cerca de 6,8 milhóes de mulheres tinham a doenga. Dentre os principais fatores de risco, destaca-se a idade avanzada para casamento, primeiro parto e menopausa, estilo de vida, condigóes ambientais e alimentares^1^.

Para reduzir os casos de cáncer de mama, mortalidade e custos, diversos países estao implementando programas de promogao da educagao em saúde as mulheres, além de estratégias de rastreamento para detecgao precoce da doenga e redugao das taxas de mortalidade^2^.

Entretanto, muitos programas de prevengao e rastreamento do cáncer de mama ainda nao atendem toda a populagao feminina. Para tanto, sao necessárias estratégias baseadas no acesso equitativo, para mitigar disparidades, principalmente entre grupos vulneráveis, como as pessoas com deficiencias^3^.

No cenário mundial, cerca de um bilhao de pessoas tem algum tipo de deficiencia, dentre elas a deficiencia auditiva. Conforme a Organizagao Mundial da Saúde, existem mais de 190 milhóes de mulheres surdas^4^. Apesar do surgimento de diversos marcos legais e políticas de inclusao no acesso ao conhecimento e servigos de saúde, as mulheres com deficiencia auditiva ainda sao excluídas e encaram barreiras de acessibilidade ao saber e tratamentos de saúde^5^.

Isso posto, abordar a temática de cáncer de mama de modo acessível a mulheres surdas é uma estratégia de inclusao social, que coaduna com o cuidado integral a este público. Diante das estratégias de promogao da saúde, a enfermagem tem papel de educador no atendimento a pessoas com surdez e a incumbencia de realizar a educagao em saúde.

Com este intuito, as tecnologias assistivas oferecem uma possibilidade educacional mais interativa, propiciando o ensino e aprendizagem e acessibilidade. Dentre os recursos tecnológicos, o vídeo educativo é uma ferramenta efetiva para o ensino de pessoas surdas, por ser compatível com a utilizagao da língua de sinais, sendo a tecnologia de maior implementagao para a educagao em saúde de pessoas surdas^6^^,^^7^.

Contudo, até o momento, há insuficiencia de evidencia na literatura de vídeo educativo com linguagem acessível para mulheres surdas acerca da prevengao e rastreamento do cáncer de mama. Assim, o estudo torna-se relevante por ofertar produto tecnológico para educagao inclusiva e acessível, com abordagem atual sobre a temática, que poderá ser utilizado para acesso a informagao de mulheres surdas, com consequente contribuigao na redugao da mortalidade por esta patologia devido ao aumento da prevengao.

Outras experiencias semelhantes retratam a prevengao e rastreamento do cáncer de mama com base em vídeos educativos, demonstrando eficácia das intervengóes sob a aprendizagem de mulheres, porém sem um direcionamento ao público com surdez^8^^-^^11^, o que fortalece o presente estudo pois possui uma abordagem inclusiva as surdas.

Cabe destacar que o vídeo poderá ser utilizado mesmo na ausencia do profissional de saúde, para comunicagao individual ou coletiva, e, uma vez que possui orientagóes claras e de forma padronizadas, autodirigidas e flexíveis, que aumentam a autonomia do telespectador, poderá ser assistido no momento, na velocidade e quantas vezes forem necessárias. Ante o exposto, este estudo teve como objetivo construir e validar vídeo educativo sobre prevengao e rastreamento do cáncer de mama para surdas.

## Materiais e Métodos

Estudo do tipo metodológico, composto pela construyo e validagáo de vídeo por especialistas no conteúdo e especialistas técnicos. Seguiram-se as recomendagóes inerentes a construyo de recursos audiovisuais. Inicialmente houve uma pré-produgáo, com construyo de sinopse, roteiro e storyboard, sendo este último a representado gráfica de uma sequéncia de agóes (análoga a narrativa em quadrinhos), com layout do produto final^12^.

A escolha dos conteúdos seguiu as recomendagóes do Instituto Nacional de Cáncer José Alencar Gomes da Silva e Ministério da Saúde brasileiro. O storyboard, com representagáo das cenas e desenhos das animagóes do vídeo foi arquitetado por designer gráfico. A preferéncia pelo vídeo com animagóes digitais ao invés de cenas com atores, reverbera no fato de que as animagóes permitem o aprimoramento estético dos desenhos e possuem menor tempo de produgáo e aparéncia atrativa^6^.

Construíram-se imagens de trés mulheres - Maria, Tereza e Luiza (personagem principal que é surda), além da enfermeira Aline. O storyboard foi produzido a partir do roteiro e serviu para definir enquadramento, posigáo e ángulo de visáo da cámera. Desenharam-se imagens a lápis, filetadas com caneta nanquim, scanneadas, colorizadas no Photoshop e montadas no InDesign. A animagáo foi realizada a partir do desenho a lápis sobre papel quadro a quadro, filetada com nanquim, fotografada, colorizado no Photoshop, editada e montada no Premiere.

A primeira versáo do storyboard foi submetida a validagáo de conteúdo por profissionais de saúde com expertise em algumas das temáticas seguintes: cáncer de mama, saúde da mulher, surdez ou vídeo educativo. Para a selegáo da amostra, considerou-se o número de seis a 20 especialistas^13^.

Enviaram-se convites a 67 profissionais com perfil elegível, porém destes, apenas 17 convidados enviaram confirmagáo de aceite para participar da pesquisa. Os profissionais foram informados acerca do prazo (20 dias) para realizar a avaliagáo do material e envio de suas recomendagóes. Após 15 dias do aceite, ou seja, cinco dias antes de finalizar o prazo, enviou-se um e-mail de lembrete. Em alguns casos foi necessário, mediante solicitagáo, estender o prazo para 25 dias, para a finalizagáo das avaliagóes. Aqueles que náo devolveram ou náo preencheram completamente os instrumentos foram excluídos. Diante disso, compuseram a amostra 11 especialistas.

Para contatar os profissionais, utilizou-se a técnica de cadeia de referéncia (bola de neve). Solicitou-se as coordenagóes de cursos de enfermagem de trés universidades, a recomendagáo de profissionais que atendiam os critérios esperados; e por meio dos primeiros participantes solicitou-se recomendagáo de outros profissionais^14^.

Consideraram-se como critérios de inclusáo: profissionais especialistas em conteúdo - profissionais da saúde, pesquisadores e docentes, com experiéncia na área de cáncer de mama, saúde da mulher, surdez ou construgáo de vídeo educativo. Considerou-se como critério de exclusáo o preenchimento incompleto do instrumento de coleta de dados.

Os profissionais foram contatados por e-mail, em que continha links do Google Forms® com Termo de Consentimento Livre e Esclarecido (TCLE); questionário de caracterizagáo dos especialistas de conteúdo; Instrumento da Validagáo de Conteúdo Educativo em Saúde (IVCES), com 18 questóes acerca dos objetivos, estrutura, apresentagáo e releváncia da tecnologia educativa^15^, além de um exemplar do storyboard em PDF.As adequagóes sugeridas pelos especialistas, foram relacionadas a exclusao de trechos e permuta de palavras para a linguagem ficar mais compreensível, clara e informativa, além de acréscimo e modificagóes nas imagens.

A versao ajustada do storyboard passou ainda pela análise crítica de sete especialistas técnicos: profissionais da área de publicidade, comunicagao, cinema, jornalismo e propaganda, design e/ou marketing. Incluíram-se profissionais com experiencia com vídeos educativos, com comunicagao audiovisual ou materiais impressos. Considerou-se como critérios de exclusao, respostas incompletas que pudessem comprometer os resultados da pesquisa.

A selegao desses profissionais ocorreu inicialmente através da busca pelo corpo docente dos cursos que possuem relagao com a temática de interesse, nos sites de universidades. Após, ocorreu igualmente uma cadeia de referencias. Os instrumentos e material foram enviados a 32 profissionais, obtendo retorno com respostas consistentes de sete especialistas, que integraram a amostra.

Os especialistas técnicos receberam um e-mail com acesso ao Google Forms® contendo o TCLE; questionário de caracterizagao dos especialistas técnicos; Instrumento para avaliar o storyboard, pelo qual se avaliou o conceito de ideia, construgao dramática, ritmo, personagens, potencial dramático, diálogo, estética, público alvo, estimativa de produgao, funcionalidade, usabilidade e eficácia; e um exemplar do storyboard em PDF.

Após avaliagao dos especialistas técnicos, os quais teceram colaboragóes a respeito dos aspectos visuais, clímax da história, cenas e recursos gráficos, desenvolveu-se a última versao do storyboard da tecnologia educativa. Posteriormente, ocorreu a produgao do vídeo pelo mesmo designer que construiu o storyboard.

A sincronia dos componentes do vídeo (animagao, legenda, narragao em áudio e em libras) foi realizada no programa Premiere. O conteúdo do vídeo foi apresentado na seguinte ordem: apresentagao inicial e créditos, 1. O que é o Cancer de Mama?; 2. Fatores de Risco para o Cancer de Mama; 3. Detecgao Precoce do Cancer de Mama; 4. Detecgao Precoce do Cancer de Mama no Sistema Único de Saúde. O vídeo todo possui narragao em Língua Brasileira de Sinais (Libras) e a intérprete ficou exposta no canto inferior direito.

Os dados foram organizados no Excel e analisados no software R, versao 4.0.4. Calcularam-se as frequencias absoluta e relativa para variáveis quantitativas. Calculou-se o Índice de Validade de Conteúdo (IVC) para cada item, a partir da proporgao de concordancia. O IVC Geral foi obtido a partir da média dos IVC. Considerou-se válido o item que obteve concordancia igual ou superior a 80%14, verificado estatisticamente, a partir da utilizagao do Teste Binomial, com significancia de 5%. O banco de dados do vídeo educativo foi armazenado no Mendeley Data^16^. É pertinente mencionar que a análise para a validagao de especialistas de conteúdo e técnicos, ocorreu separadamente.

A pesquisa foi aprovada pelo Comite de Ética da Universidade da Integragao Internacional da Lusofonia Afro-Brasileira (n. ° 3.936.677/2020) e os participantes consentiram participar por meio do TCLE.

## Resultados

O storyboard foi composto por 21 páginas, com 78 imagens autorais. O vídeo completo possui

17 minutos e 12 segundos, com apresentagáo inicial, créditos e quatro blocos de conteúdo, com acessibilidade em LIBRAS.

Os especialistas de conteúdo eram enfermeiros, a maioria doutores 63,63% (n=7), com experiencia em saúde da mulher 72,63% (n=8), cáncer de mama 63,63% (n=7) e construyo de vídeos 45,45% (n=5). Houve concordancia mínima de 90,90% (n=10) quanto aos objetivos, estrutura e apresentagáo e releváncia, e, 90,90% (n=10) concordaram que o vídeo estimula o aprendizado, contribui para o conhecimento na área e desperta interesse pelo tema; está adequado ao processo de ensino e aprendizagem, incentiva mudanza de comportamento, linguagem interativa, permitindo envolvimento ativo no processo educativo e tema atual (Tabela 1).

**Tabela 1:** Concordancia dos juízes acerca dos objetivos, estrutura, apresentaáo e relevancia. Redenáo, CE, Brasil, 2021

Variáveis	Parcialmente adequado	Adequado	IVC	p*
	%(n)	%(n)		
Objetivos: propósitos, metas ou finalidades				
Contempla o tema proposto	18,20(2)	81,80(9)	1,00	1
Adequado ao processo de ensino e aprendizagem	0,00(0)	90,90(10)	0,90	0,9141
Esclarece dúvidas sobre o tema abordado	18,20(2)	72,70(8)	0,90	0,9141
Proporciona reflexao sobre o tema	18,20(2)	72,70(8)	0,90	0,9141
Incentiva mudanza de comportamento	0,00(0)	90,90(10)	0,90	0,9141
Geral	10,90(6)	81,80(45)	0,92	0,997
Estrutura e apresentado: organizado, estrutura, estratégia, coerencia e suficiencia				
Linguagem adequada ao público-alvo	18,20(2)	72,70(8)	0,90	0,9141
Linguagem apropriada ao material educativo	27,30(3)	63,60(7)	0,90	0,9141
Linguagem interativa, permitindo envolvimento ativo no processo educativo	0,00(0)	90,90(10)	0,90	0,9141
Informales corretas	54,50(6)	45,50(5)	1,00	1
Informales objetivas	18,20(2)	72,70(8)	0,90	0,9141
Informales esclarecedoras	18,20(2)	72,70(8)	0,90	0,9141
Informales necessárias	9,10(1)	81,80(9)	0,90	0,9141
Sequencia lógica das ideias	18,20(2)	72,70(8)	0,90	0,9141
Tema atual	9,10(1)	90,90(10)	1,00	1
Tamanho do texto adequado	18,20(2)	72,70(8)	0,90	0,9141
Geral	19,10(21)	73,60(81)	0,92	0,999
Relevancia: significancia, impacto, motivado e interesse				
Estimula o aprendizado	0,00(0)	90,90(10)	0,90	0,9141
Contribui para o conhecimento na área	0,00(0)	90,90(10)	0,90	0,9141
Desperta interesse pelo tema	0,00(0)	90,90(10)	0,90	0,9141
Geral	0,00(0)	90,90(30)	0,90	0,9732

*Teste binomial. Fonte: dados da própria pesquisa, 2021

Quanto aos especialistas técnicos, a maioria era da Comunicado Social - Jornalismo 42,90% (n=3) e doutores 71,50% (n=5), com experiencia com comunicado audiovisual 100,00% (n=7) e materiais impressos 85,80% (n=6). Estes reportaram que a organizado do vídeo é atraente, dinámico e didático, a variedade de cenas é apropriada e os recursos gráficos foram bem colocados na anatomia humana, deixando claro a sintomatologia da doenga (Tabela 2).

**Tabela 2:** Valor do índice de validade de conteúdo por itens individuais do conteúdo por juízes especialistas em aparencia. Fortaleza, CE, Brasil, 2018

Parámetros/ Questionamentos	Sim		p*
	N	%	
Conceito de ideia			
O storyboard é adequado ao objetivo que se propoe de capacitar mulheres surdas	7	100,0	1
sobre o cáncer de mama?			
A ideia auxilia a aprendizagem?	7	100,0	1
A ideia é acessível?	7	100,0	1
O storyboard é útil?	7	100,0	1
O storyboard é atrativo?	7	100,0	1
Construyo dramática (abertura, conflito, desenvolvimento, clímax, final)			
Ponto de partida do storyboard tem impacto?	6	85,7	0,7903
Com o desenvolvimento do storyboard o interesse cresce?	6	85,7	0,7903
Número de cenas é suficiente?	5	71,4	0,4233
O tempo de duraáo é suficiente?	6	85,7	0,7903
O storyboard tem apresentaáo agradável?	7	100,0	1
Ritmo (evoluao dos momentos dramáticos, tipos de cena)			
Existe uma atenáo crescente, com curvo dramática ascendente?	6	85,7	0,7903
O ritmo é cansativo?	3	42,9	0,1480
Há dinamismo dos ambientes?	5	71,4	0,4233
As formas de apresentaáo das cenas sáo adequadas?	7	100,0	1
Personagens (motivado, credibilidade, interaao)			
O perfil das personagens é original?	6	85,7	0,7903
Os valores das personagens tem consistencia?	7	100,0	1
Potencial dramático			
É desenvolvida uma expectativa?	5	71,4	0,4233
Diálogos (tempo dramático)			
No diálogo cada intervengo motiva outra?	5	71,4	0,4233
Há acelerado da aáo até o ponto culminante do clímax da história?	3	42,9	0,1480
Estilo visual (estética)			
Existem muitas repetioes de cenário/ambiente?	1	14,3	0,7903
As imagens sáo adequadas?	7	100,0	1
A estrutura geral é criativa?	7	100,0	1
Público referente			
O conteúdo de interesse (educaáo sobre o cáncer de mama) tem relaáo direta com o público alvo (mulheres surdas)?	7	100,0	1
Funcionalidade: refere-se as fundes que sao previstas pelo vídeo educativo			
O vídeo, como está no storyboard, propoe-se a educar as mulheres surdas sobre o cáncer de mama?	7	100,0	1
O vídeo é capaz de gerar resultados positivos?	7	100,0	1
Usabilidade: refere-se ao esforo necessário para usar o vídeo, bem como o julgamento individual desse uso por um conjunto explícito ou implícito de usuários			
É fácil aprender os conceitos utilizados e suas aplicares?	7	100,0	1
Permite que a mulher compreenda os fatores de risco e autocuidados apresentados, sendo fácil compreender e realizar?	7	100,0	1
Fornece ajuda de forma clara?	7	100,0	1
Fornece ajuda de forma completa?	6	85,7	0,7903
Fornece ajuda sem ser cansativo?	7	100,0	1

*Teste binomial. Fonte: arquivos da própria pesquisa, 2021

A maior parte dos critérios foram bem avaliados pelos especialistas, em especial o conceito de ideia e funcionalidade, aprovados em todas suas estratificagóes pelo conjunto total de especialistas. As discordancias com relagao ao storyboard foram poucas, relacionadas ao número de cenas 28,60% (n=2), dinamismo do ambiente 28,60% (n=2), na expectativa e no diálogo de cada 28,60% (n=2) (Tabela 3).

**Tabela 3:** Avaliagáo do storyboard pelos especialistas técnicos. Redengáo, CE, Brasil, 2021

Critérios	Excelente %(n)	Muito bom %(n)	Bom %(n)	Regular %( n)	p*
Avaliagáo da ideia	57,10(4)	42,90(3)	-	-	1,0
Avaliagáo da construgáo dramática	28,60(2)	57,10(4)	14,30(1)	-	1,0
Avaliagáo do ritmo	42,90(3)	28,60(2)	14,30(1)	14,30(1)	0,7903
Avaliagáo das personagens	57,10(4)	42,90(3)	-	-	1,0
Avaliagáo do potencial dramático	28,60(2)	28,60(2)	28,60(2)	14,30(1)	0,7903

*Teste binomial. Fonte: arquivos da própria pesquisa, 2021

Os itens mais aceitos pelos especialistas, considerados excelentes, foram a avaliagao de ideia 57,10% (n=4) e avaliagao das personagens 57,10% (n=4). Os itens de maior desacordo, considerados regular por um especialista, foram a avaliagao de ritmo 14,30% (n=1) e a avaliagao do potencial dramático 14,30%(n=1).

## Discussao

O vídeo educativo foi considerado válido para permear o ensino e aprendizagem sobre o tema e contribuir com agóes de autocuidado e promogao da saúde as mulheres surdas. Nesse contexto, é imprescindível a inclusao social da comunidade surda, dado que possuem linguagem própria, desconhecida pela maioria das pessoas, inclusive profissionais de saúde^17^.

Destaca-se que os meios de comunicado audiovisuais nem sempre implementam tecnologías para a compreensáo das pessoas surdas, bloqueando o recebimento de informagóes^18^. Assim, o presente estudo buscou acolher uma necessidade de mulheres surdas, a respeito do cancer de mama. Outros estudos com público surdo compartilham da inclusao de LIBRAS no vídeo educativo^6^^,^^19^.

No estudo em tela, utilizou-se o recurso de animagáo, com objetivo de apresentar as informales de forma atrativa. Diversos estudos também utilizaram essa opgáo de vídeo^6^^,^^20^^,^^21^. Estudo recente acerca da efetividade de vídeo educativo com recurso de animado para surdos identificou que esta tecnologia foi efetiva no aumento do conhecimento e habilidade de pessoas com surdez acerca da ressuscitado cardiopulmonar^22^. Dessa forma, é pertinente o emprego desse perfil de vídeo educativo para educado em saúde da populado.

Na validado de conteúdo, os especialistas concordaram quanto a clareza e sequéncia lógica das ideias. Todavia, houveram sugestóes de ajustes na linguagem. A possibilidade de ajustar detalhes no vídeo antes de divulga-lo ao público-alvo é uma etapa importante para garantir que o material seja efetivo e adequado. Outros estudos de validado corroboram que as recomendagóes de especialistas tornam a linguagem do vídeo mais e clara e objetiva^20^^,^^23^.

Vale destacar que também se buscou a avaliagáo por especialistas em vídeo. Essa foi uma estratégia pertinente, dado que muitos aspectos visuais, clímax da história, cenas e recursos gráficos foram apontados pelos juízes técnicos, o que diverge de outros estudos de validagáo com pessoas surdas, que utilizam apenas especialistas na temática^7^^,^^19^.

As recomendagóes dos juízes contribuíram com as associagóes entre a realidade do público-alvo e o caráter educador do vídeo, para criar um efeito emocional. Esse é um aspecto relevante, uma vez que a insergáo de elementos que evocam os sentidos, prende a atengáo do telespectador até o final, contribuindo com o interesse pelo assunto^24^^,^^25^.

Os especialistas técnicos sugeriram colocar um aparelho auditivo na personagem principal, dando mais representatividade ao público alvo, a medida que Luiza demonstra satisfagáo e compreensáo das informagóes compartilhadas pela enfermeira Aline.

Nos achados da produgáo de outro vídeo educativo^26^, os autores descrevem que na cena final, em que os diversos profissionais envolvidos no atendimento realizam uma reuniáo final, para o melhor entendimento do significado esperado para o vídeo. De maneira semelhante, os especialistas técnicos sugeriram que ao final do vídeo as personagens reaparecessem, para demostrar a satisfagáo com relagáo ao aprendizado adquirido, proporcionando um clímax final a história do vídeo educativo.

Pelo fato do público-alvo ser mulheres surdas, uma característica diferencial e de suma importancia foi aplicada no vídeo, a gravagáo das cenas com uma intérprete de LIBRAS, de modo que a comunicagáo do conteúdo fosse efetiva. Em muitos cenários, inclusive de atengáo a saúde, na auséncia de intérprete, algumas pessoas, para se fazer entender pelos surdos, utilizam dramatizagóes, mímicas, datilologias, escrita etc. para se comunicar. Contudo, existe uma gama de alternativas no ambito das tecnologias assistivas, que podem auxiliar no processo de aprendizagem de surdos^27^.

Estudo consultado destaca como vantagem da iniciativa de tecnologias assistivas no contexto do cancer de mama para surdas, o conhecimento, em que se identificou que 79% de mulheres surdas do grupo de intervengáo obtiveram por meio de vídeos educativos, informagóes que náo receberam de um médico. Isso pode estar relacionado ao fato de que os vídeos dizem respeito a um programa gravado em cinco capítulos que incluía a língua de sinais americana, legendas em inglés, locugáo em inglés e foco em imagens visuais (fotografias, videoclipes, animagáo e dramatizado) e folheto^28^.

Outra tecnologia assistiva que demostrou vantagem, foi o audiotexto sobre cáncer de mama, aplicado a mulheres com deficiéncia visual, no qual foram abordados anatomia da mama, conceito, métodos de diagnóstico, auto palpado, sinais e sintomas, fatores de risco, prevendo, rastreamento e tratamento. A presente tecnologia assistiva também foi considerada adequada em dois países, Brasil e Portugal, onde as participantes a avaliaram positivamente^29^.

De modo semelhante, porém desenvolvida para mulheres sem deficiéncia, uma intervendo educativa com quatro vídeos informativos breves, com durado de 3 a 5 minutos, foi aplicada com mulheres após o resultado da mamografia, nos quais os conteúdos variavam com base no risco de cáncer de mama da mulher (alto ou médio) e o resultado de densidade mamográfica (maior ou menor). Demonstrou-se que o grupo intervendo que assistia aos vídeos informativos, além de receber uma carta legalmente exigida sobre o resultado da densidade mamográfica, teve maior melhora em seu conhecimento de sua densidade mamográfica pessoal, em comparado ao grupo controle que teve acesso somente uma carta informativa obrigatória por lei^11^.

Nesse cenário, percebe-se a importancia da produdo de materiais educativos para atender as demandas de saúde, em específico para as consultas de Enfermagem, pois oferece conteúdo apropriado, suficiente para atender as necessidades do público-alvo, associado a aplicado de texto compreensível, escrito de maneira atrativa e objetiva^6^.

Destaca-se como limitado do estudo, a falta da validado semántica com o público alvo. Porém, o estudo traz implicagóes positivas para o avanzo do conhecimento científico na enfermagem, com expansáo da ferramenta tecnológica em formato de vídeo educativo para a educado em saúde inclusiva, de forma a possibilitar o acesso a informagóes baseadas em evidéncias, de forma clara e acessível a mulheres surdas.

## Conclusao

O vídeo educativo sobre prevengáo e rastreamento do cáncer de mama para surdas foi construído e validado pelos especialistas em conteúdo e aspectos técnicos. A tecnologia permitirá que mulheres surdas possam exercer o autocuidado com orientagóes precisas e claras, pois a comunicagáo empregada dispóe além do áudio, da legenda na língua portuguesa para aquelas que sabem ler, e da tradugáo em LIBRAS por uma intérprete. Como complementar a compreensáo das mensagens do vídeo, tém-se ainda o incremento de imagens animadas explicativas, dispondo assim de diversos elementos lúdicos que promovem a acessibilidade das mulheres surdas a informagóes confiáveis, sendo assim instruídas sobre a prevengáo e rastreamento do cáncer de mama.
